# Updated Three-Stage Model for the Peopling of the Americas

**DOI:** 10.1371/journal.pone.0003199

**Published:** 2008-09-17

**Authors:** Connie J. Mulligan, Andrew Kitchen, Michael M. Miyamoto

**Affiliations:** 1 Department of Anthropology, University of Florida, Gainesville, Florida, United States of America; 2 Department of Zoology, University of Florida, Gainesville, Florida, United States of America; University of Utah, United States of America

## Abstract

**Background:**

We re-assess support for our three stage model for the peopling of the Americas in light of a recent report that identified nine non-Native American mitochondrial genome sequences that should not have been included in our initial analysis. Removal of these sequences results in the elimination of an early (i.e. ∼40,000 years ago) expansion signal we had proposed for the proto-Amerind population.

**Methodology/Findings:**

Bayesian skyline plot analysis of a new dataset of Native American mitochondrial coding genomes confirms the absence of an early expansion signal for the proto-Amerind population and allows us to reduce the variation around our estimate of the New World founder population size. In addition, genetic variants that define New World founder haplogroups are used to estimate the amount of time required between divergence of proto-Amerinds from the Asian gene pool and expansion into the New World.

**Conclusions/Significance:**

The period of population isolation required for the generation of New World mitochondrial founder haplogroup-defining genetic variants makes the existence of three stages of colonization a logical conclusion. Thus, our three stage model remains an important and useful working hypothesis for researchers interested in the peopling of the Americas and the processes of colonization.

## Introduction

We recently published a three stage model for the peopling of the Americas [1]. Specifically, we proposed that a recent, rapid expansion into the Americas was preceded by a long period of population stability in greater Beringia by the proto-Amerind population after divergence from their ancestral Asian population. We used two complementary coalescent methods, Bayesian skyline plot [2] and isolation-by-migration [3] analyses, to estimate past population growth patterns in Native American populations and to estimate a New World founder effective population size. We explicitly incorporated archaeological, geological, and paleoecological constraints into our analyses to enhance the anthropological relevance of the results and to provide a comprehensive model for the initial settlement of the Americas.

Fagundes et al. [4] have published a re-analysis of the data we used in developing our three stage model for the peopling of the Americas [1]. Specifically, they identified nine mitochondrial coding region sequences that we assumed were Native American sequences, but instead are likely to derive from Asian or European individuals. Fagundes et al. are correct in this assessment, i.e. five sequences were reclassified as Asian after their publication as Native American sequences [5] and four sequences were mistakenly included in our original study. The effect of removing these sequences from the Bayesian skyline plot analysis is that the suggestion of an early expansion event in the skyline plot is no longer apparent, a finding that we have reconfirmed by re-running our original dataset without these nine genomes. It appears that the non-Native American sequences introduced additional variation that created an expansion signal that does not exist in an analysis of only Native American sequences.

In light of these facts, we have now analyzed the largest dataset of Native American mitochondrial coding genomes using publicly available sequences (n = 148; [6]) in a Bayesian skyline plot analysis. We also provide an estimate for the duration of the period of population isolation required for the generation of New World founder haplogroup-defining variants. As in our previous analysis, we evaluate the significance of our results in concert with other non-genetic data.

## Results

**Figure 1:**
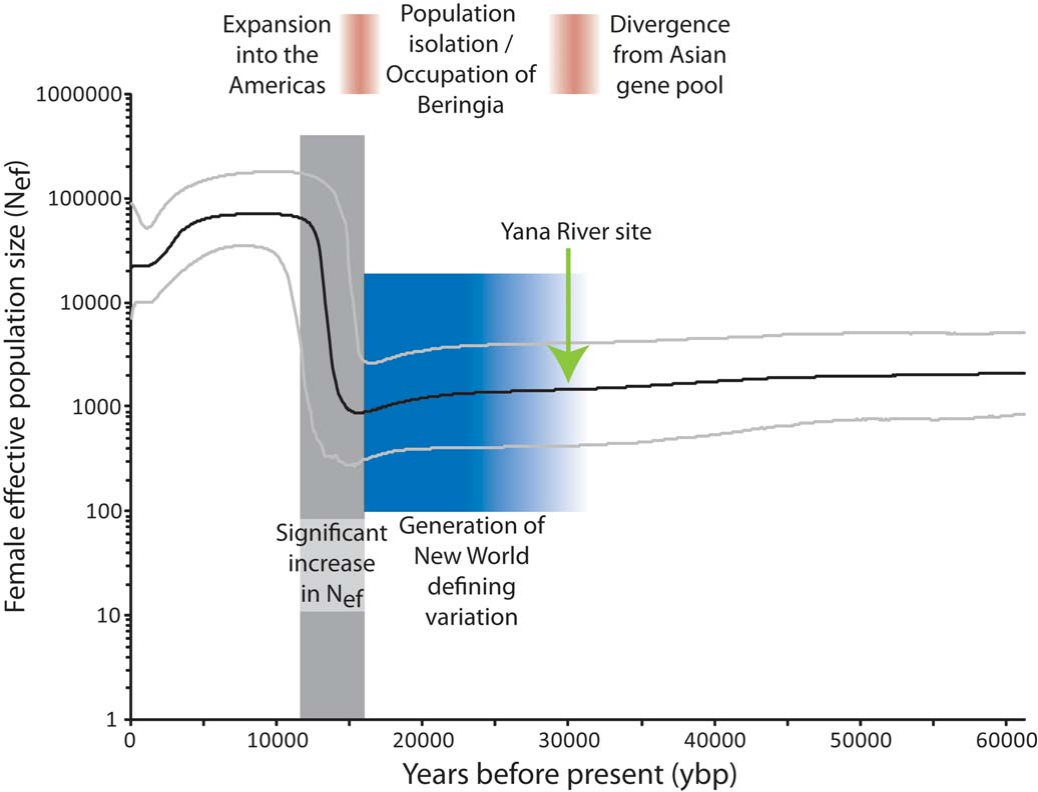
Bayesian skyline plot of 148 Native American mitochondrial coding genome sequences. The curve plots median N_ef_ with 95% credible intervals indicated by light gray lines. The shaded gray box highlights the significant increase of N_ef_ during the colonization of the Americas 16–12 kya. The blue box depicts the calculated time required for the generation of New World defining mitochondrial variants and its shaded region represents the variation in these estimates, i.e. 7–15 thousand years before entry to the New World (see Table 1). The green arrow identifies the date of the Yana River site of human occupation in western Beringia [21].

We use a Bayesian skyline plot to visually illustrate changes in Native American female effective population size (N_e_) over time. Bayesian skyline plots assume a single migration event, which makes the approach ideal for questions concerning the peopling of the Americas since it is generally agreed that there was a single migration [7]. Our new skyline plot (Fig. 1) strongly supports a large population expansion (∼1.8 orders of magnitude, or 80-fold) that occurred ∼16–12 thousand years ago (kya). This timing suggests an entry to the New World that was coincident with the retreat of the North American ice sheets, i.e. the opening of an ‘ice-free corridor’ ∼17–14 kya [8], [9]. Immediately before this expansion, there is a small drop in effective population size ∼17–16 kya (this is an insignificant change, as judged by the overlap in 95% confidence intervals at the beginning and end of the population decrease), possibly corresponding to a population bottleneck prior to entry to the Americas. Before 17 kya, the skyline plot is flat with no evidence of the early (∼40 kya) population expansion we reported previously [1]. The absence of an early expansion signal in the skyline plot may simply indicate that divergence of proto-Amerinds from the Asian gene pool was not accompanied by significant population growth. These results are highly consistent with our earlier analysis of only 20 Native American mitochondrial coding genomes [10], in support of theoretical expectations by Felsenstein [11] that increasing sample size is an inefficient way to improve the accuracy of maximum likelihood estimations from coalescent analyses of population genetic data.

Our new analysis (with non-Native American sequences eliminated and more Native American sequences added) shows a larger population increase (80-fold vs 16-fold) over a smaller period of time (16–12 kya vs 16–9 kya) relative to our previous analysis that inadvertently included non-Native American sequences [1]. The non-Native American sequences likely introduced additional variation that artificially increased N_e_ prior to the expansion. Thus, we can estimate a new N_e_ for the New World founding population of 1,800 (this number is multiplied by two since the skyline plot only estimates the female effective population size). This number is closer to our previous isolation-with-migration (IM)-based estimate of 1,200 [1] and thus reduces the variation around our estimate of the size of the founding population to ∼1,000–2,000 effective individuals.

Prior to entry to the New World, we propose a period of isolation. A valid question remains - How long was the period of isolation? In the absence of a biphasic skyline plot, we can calculate first approximations of the time necessary to generate the defining variants for the New World mithochondrial founding haplogroups. All New World mitochondrial sequences cluster in five monophyletic clades, representing founding haplogroups that are differentiated from non-New World haplogroups by the presence of specific, defining genetic variants. The variants that occur on the branch leading to each New World founding haplogroup represent variation that evolved prior to expansion into the Americas whereas variation within each founding haplogroup, i.e. nucleotide diversity within a haplogroup, represents variation that evolved after entry to the Americas – we are interested in the variation that occurred prior to entry into the Americas. There is strong consensus on the number of New World founding haplogroup-defining variants, including both coding and non-coding hypervariable regions I and II (HVRI+II) variants [5], [12]. However, there is a wide range of substitution rates that have been estimated for both coding and non-coding variants [13]–[17]. Fagundes et al. [4], [18] tend to favor the slower substitution rates whereas we generally favor the faster substitution rates, particularly for coding variants since a faster rate (∼1.7×10^−8^ substitutions/site/year) has been confirmed using two independent approaches [13], [16]. However, to be complete since there is ongoing debate about the correct calculation of substitution rates most recently [19], [20], we present a series of estimates based on coding and HVRI+II variants using both fast and slow substitution rates (Table 1). As is evident from the calculations, there is a wide range of estimates for the time necessary to generate the New World defining variants, i.e. averages range from ∼6,000 to ∼25,000 years. By averaging across coding and non-coding variants and including fast and slow substitution rates, we report a range of ∼7–15 thousand years. This estimate suggests that Amerind ancestors may have experienced a period of isolation lasting at least 7–15 thousand years prior to their expansion into the Americas (see the blue box in Fig. 1).

**Table 1:** Estimates of time necessary to generate the mitochondrial genome variants that define New World founding haplogroups.

Founding haplogroups based on coding variants a	# defining variants b	Time necessary to generate haplogroup defining coding variants using a fast substitution rate (years) c	Time necessary to generate haplogroup defining coding variants using a slow substitution rate (years) c
H'grp A2	2	7,616	10,276
H'grp B2	5	19,040	25,690
H'grp C1b	1	3,808	5,138
H'grp C1c	2	7,616	10,276
H'grp C1d	1	3,808	5,138
H'grp D1	1	3,808	5,138
H'grp X2a	3	11,424	15,414
Average (coding)		8,160	11,010

Founding haplogroups based on HVRI+II variants a		Time necessary to generate haplogroup defining HVRI+II variants using a fast substitution rate (years) c	Time necessary to generate haplogroup defining HVRI+II variants using a slow substitution rate (years) c
H'grp A2	3	9,066	37,053
H'grp D1	1	3,022	12,053
H'grp X2a	2	6,044	24,702
Average (HVRI+II)		6,044	24,702
Average (coding and HVRI+II)		7,525	15,118

a The total number of defining variants for a single founding haplogroup (H'grp) was used in each calculation. Haplogroups B2 and C1b–d do not have defining HVRI or HVRII variants and were therefore not used in the HVRI+II calculations. Averages were calculated for coding and HVRI+II variants separately as well as an average of the total number of estimates within each substitution rate.

b The number of defining variants for New World founding haplogroups was determined by Bandelt et al. [5] and Tamm et al. [12].

c Substitution rates were as follows: Coding/Fast = 1.7×10^−8^ substitutions/site/year→3,808 years/mutation [13], [16]; Coding/Slow = 1.26×10^−8^ substitutions/site/year→5,138 years/mutation [17]; HVRI+II/Fast = 4.7×10^−7^ substitutions/site/year→3,022 years/mutation [15]; HVRI+II/Slow = 1.15×10^−7^ substitutions/site/year→12,351 years/mutation [14].

## Discussion

Our proposal for a three stage model for the peopling of the Americas remains essentially unchanged despite the modifications to the skyline plot described above. The three stages remain; 1) divergence of Amerind ancestors from the Asian gene pool, 2) prolonged period of isolation, lasting at least 7–15 thousand years, during which time genetic variants specific to and present throughout the New World were generated, and 3) rapid expansion into the Americas ∼16 kya concomitant with a large population increase. The existence of mitochondrial New World-defining variants that are widespread throughout the Americas has been noted in numerous publications most recently [6], [12] and indicates that there must have been a period of isolation during which time these variants arose. The idea of a period of population isolation prior to expansion into the Americas was first mentioned by Bonatto and Salzano [14] and most recently supported by Tamm et al. [12]. Thus, divergence from the Asian gene pool and entry into the Americas were separated by this period of isolation, making the existence of three stages a logical conclusion.

In our previous study, we suggested that the period of isolation occurred during occupation of greater Beringia [1]. The fact that Beringia is now inundated may explain why no archaeological evidence of human occupation has been found, although greater Beringia encompasses such a vast territory that more terrestrial archaeological sites may yet be discovered. The documentation of human occupation at the Yana River site ∼30 kya [21] provides independent support for the presence of humans in greater Beringia as early as 30,000 years ago [22] and strengthens our proposal of a Beringian occupation from ∼30–16 kya. Furthermore, multiple fossil sites document the presence of large mammals in Alaska and Siberia [23]–[25]. Fossil pollen and plant microfossils from eastern Beringia indicate a productive, dry grassland ecosystem [26] suggesting the entire range of Beringia was capable of supporting a large mammal fauna. Archaeological evidence and ethnographic analogy both suggest that Amerind ancestors in Beringia were skilled hunters who relied upon megafauna for sustenance and likely extended their hunting ranges in response to demographic changes in the large mammal population [27]. Thus, it is highly probable that humans inhabited the central part of greater Beringia, i.e. Beringia, for an extended period of time. In fact, the first published Bayesian skyline plot focused on the Beringian steppe bison (using 169 ancient DNA sequences and 22 modern sequences) and revealed a sharp population decline beginning ∼30 kya [2] leading us to suggest that Beringian populations of humans may have been associated with the decline in steppe bison.

In conclusion, our three stage model remains an important and useful working hypothesis for researchers interested in the peopling of the Americas and the processes of colonization. We believe that divergence from the ancestral gene pool and expansion into a new territory were not simultaneous events, as is often assumed in models of population demographic history. Specifically, movement from Asia to the New World was interrupted by an extended period of population isolation and stability. Entry into the New World was mediated by a population of 1,000–2,000 effective individuals. The relevance of our model is due to its reliance on a synthetic approach that combines genetic data with multiple sources of anthropological and paleoenvironmental information. As a working hypothesis, our model is predictive. In particular, it predicts that key archaeological sites await discovery under the Bering Sea.

## Materials and Methods

A dataset of 148 human mitochondrial coding genomes was assembled from the publicly available sequences used by Achili *et al.* [6] and then aligned as described in Kitchen *et al.* [1]. Bayesian skyline plots [2] of the aligned coding genomes were used to estimate changes in Amerind N_ef_ over time by providing highly parametric, piecewise estimates of N_ef_. In these analyses, estimates of τ (N_ef_×generation time) were converted to N_ef_ by dividing by a generation time of 20 years, following convention [3]. Using a generation time of 25 years decreases N_ef_ estimates by 20%, but does not affect the time estimates. Skyline plots were generated using the program BEAST v1.4 (http://beast.bio.ed.ac.uk). These BEAST analyses relied on the same coalescent and substitution models and run conditions as Kitchen *et al.* [10]. Markov chains were run for 100,000,000 generations and sampled every 2,500 generations with the first 10,000,000 generations discarded as burn-in.
